# Production of Mannosylerythritol Lipids (MELs) to be Used as Antimicrobial Agents Against *S. aureus* ATCC 6538

**DOI:** 10.1007/s00284-020-01927-2

**Published:** 2020-03-02

**Authors:** Chiara Ceresa, Simon Hutton, Marta Lajarin-Cuesta, Robert Heaton, Iain Hargreaves, Letizia Fracchia, Mayri A. Díaz De Rienzo

**Affiliations:** 1 Department of Pharmaceutical Sciences, Università del Piemonte Orientale “A. Avogadro”, 28100 Novara, Italy; 2 School of Pharmacy and Biomolecular Sciences, Liverpool John Moores University, James Parsons Building 10.05C, Byrom Street, Liverpool, L3 3AF UK

## Abstract

Antimicrobial resistance (AMR) is a current major health issue, both for the high rates of resistance observed in bacteria that cause common infections and for the complexity of the consequences of AMR. Pathogens like *Staphylococcus aureus*, *Pseudomonas aeruginosa*, *Klebsiella pneumoniae*, *Mycobacterium tuberculosis* among others are clear examples of antibiotic-resistant threats. Biosurfactants have recently emerged as a potential new generation of anti-adhesive and anti-biofilm agents; mannosylerythritol lipids (MELs) are biosurfactants produced by a range of fungi. A range of structural variants of MELs can be formed and the proportion of each isomer in the fermentation depends on the yeast used, the carbon substrate used for growth and the duration of the fermentation. In order to allow assessment of the possible functions of MELs as antimicrobial molecules, small quantities of MEL were produced by controlled fermentation. Fermentations of the yeast *Pseudozyma aphidis* using rapeseed oil as a carbon source yielded up to 165 g_MELs_/kg_Substrate_. The MELs formed by this strain was a mixture of MEL-A, MEL-B, MEL-C and MEL-D. The MELs produced were tested against *S. aureus* ATCC 6538 on pre-formed biofilm and on co-incubation biofilm experiments on silicone discs; showing a disruption of biomass, reduction of the biofilm metabolic activity and a bacteriostatic/bactericidal effect confirmed by a release of oxygen uptake $p_{{{\text{O}}_{2} }}$, the reduction of citrate synthase activity and scanning electron microscopy. The results show that MELs are promising antimicrobial molecules for biomedical technological applications that could be studied in detail in large-scale systems and in conjunction with animal tissue models.

**Electronic supplementary material:**

The online version of this article (10.1007/s00284-020-01927-2) contains supplementary material, which is available to authorized users.

## Introduction

Antimicrobial resistance (AMR) is one of the principal concerns of the public health worldwide, that in recent years have only worsened [1]. This is due to a combination of factors, varying from the overuse of broad-spectrum antibiotics in agricultural and food industries, to a lack of public understanding of appropriate usage of prescribed antibiotics [2, 3]. For these reasons, developing alternative antibiotics at a faster rate than microorganisms can establish resistance is essential [4, 5]. A prevalent factor influencing this is the role that biofilms play on infections and subsequent antibiotic resistance [1]. This ability of pathogenic biofilm survival within highly concentrated antibiotic environments has been denoted as “recalcitrance”, a characteristic that leads to treatment failure and infection recurrence [5]. As classic methods of antibiotic resistance become more established, it has become clearer that their role in the ability of biofilms to withstand antibiotics is reduced [6–8]. In the recent years biosurfactants have emerged as effective antimicrobials and anti-adhesive molecules [9], biosurfactants are amphiphilic compounds produced within microbial living spaces or excreted extracellularly, constituted of a hydrophilic and hydrophobic domain [10]. This variability has driven a recent interest in the industrial applications of biosurfactants, mainly as an alternative to chemical surfactants. As a comparison, biosurfactants have demonstrated significant structural diversity, lower toxicity and production conditions and higher biodegradability, foaming ability, selectivity and specific activity at extreme conditions [11, 12]. Pharmaceutical applications include antimicrobial, anti-cancer and anti-biofilm properties, the former and latter promoting an alternative approach to synthetic antibiotics [13–15].

**Fig. 1:**
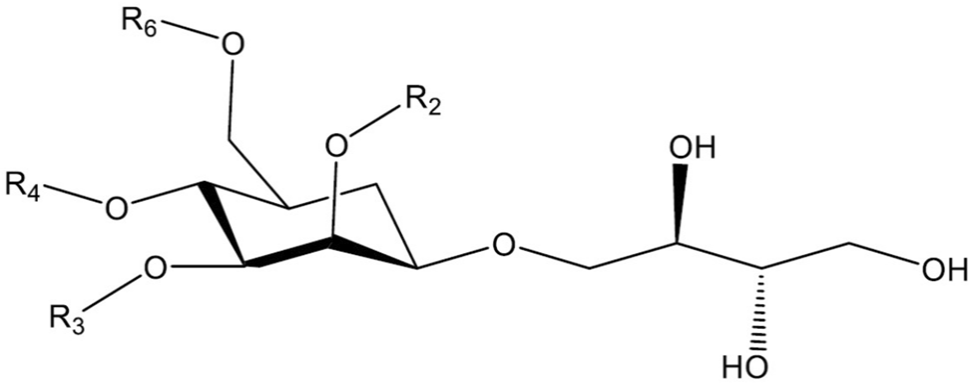
Molecular structure of mannosylerythritol lipids. The length and saturation of the fatty acid residues depend on the substrate and microorganism used. MEL-A → R1: Acetyl, R2: Acetyl; MEL-B → R1: Acetyl, R2: H; MEL-C → R1: H, R2: Acetyl; MEL-D → R1: H, R2: H

Among the biosurfactants that exhibit these properties, the mannosylerythritol lipids (MELs) and rhamnolipids have especially been investigated [16]. Biosurfactants consisting of a fatty acid and/or an acetyl group as the hydrophobic moiety and a 4-*O-*β-d-mannopyranosyl-*meso*-erythritol as the hydrophilic group can be categorized as a MEL (Fig. 1) [10, 17]. There is a clear consensus on the antimicrobial and anti-adhesive properties potential displayed by the more commonly investigated biosurfactants, such as rhamnolipids and sophorolipids, tested against both planktonic and biofilm physiologies [9, 18–20]. In this work, the antimicrobial effects of MELs on *Staphylococcus aureus* biofilms were identified; having a positive impact on the disruption of biomass, biofilm metabolic activity and relative biofilm oxygen uptake.

## Materials and Methods

### Bacterial Strains, Growth Conditions and Chemical Analysis

*Staphylococcus aureus* ATCC 6538, was cultivated in tryptic soy broth (TSB) 50% + glucose (10 g/L) and stored at − 20 °C until further use in the biofilm experiments. *Pseudozyma aphidis* (ATCC 32657) stock cultures were incubated for 2 days on potato dextrose agar (PDA) slants at 30 °C, stored at 4 °C and renewed monthly. For the seed culture glucose (30 g/L), yeast extract (1 g/L), NH_4_NO_3_ (1 g/L) and KH_2_PO_4_ (0.3 g/L) were used for the fermentation process rapeseed oil (80 g/L), NaNO_3_ (2 g/L), KH_2_PO_4_ (0.20 g/L), MgSO_4_·7H_2_O (0.20 g/L) and yeast extract (1 g/L) were used.

The fermentation parameters were set at 30 °C, 180 rpm and an aeration rate of 1 volume per volume per minute (vvm). For the fed-batch fermentation, the same procedure was followed, after 2 days the culture was fed with 200 g of rapeseed oil, and on day 5 another 288 g were added. After 7 days the fermentation product was then ready to be harvested with a yield of 165 g_MELs_/kg_Substrate_. MELs were purified from supernatants of *P. aphidis* as described previously [21]. Briefly, the MEL-enriched phase was dissolved in ethyl acetate and the organic phase was filtered on Na_2_SO_4_ and evaporated until a brown sticky oil remained (crude MEL mix). This crude mix was submitted to a series of washing steps with n-hexane, methanol and water, followed by vacuum evaporation resulting in an MEL mix free from apolar glycolipids and residual free fatty acids (FFAs). This purified extract was further used as a reference mixture containing MEL-A, -B, -C and -D for thin-layer chromatography (TLC) analyses.

MEL extracts were analysed by TLC on silica gel 60 TLC plates in two‑step visualization using dichloromethane–acetone (60:40) as the solvent system. Afterwards, the isolated compounds on the TLC plates were located by charring at 110 °C for 5 min after spraying the plates with an orcinol reagent with sulfuric acid (0.1% orcinol in 5% H_2_SO_4_). Glycolipids containing hexoses appear as purple spots, triglycerides as yellow to brownish spots. To visualize FFAs, the TLC plate was placed under iodine vapours. For the ESI–MS analysis a Waters Micromass LCT TOF (time of flight mass analyser), operated in negative ion mode was used, the extracts were diluted in methanol and the ionization parameters were set at 400–1000 m/*z*, a desolvation temperature of 200 °C was applied together with a desolvation gas flow (L/h) of 694 and a capillary voltage of 3000 V.

### Inhibition of Biofilm Formation

Medical-grade silicone discs (SEDs) (TECNOEXTR) of 10 mm in diameter and 1.5 mm in thickness were cleaned and sterilized as previously described [22]. Briefly, SEDs were dipped in a 1.4% (v/v) RBSTM 50 solution, sonicated for 5 min at 60 kHz and rinsed twice in Milli-Q water. Discs were, then, dipped in methanol (99%), sonicated and rinsed as before. Silicone was autoclaved and dried at 37 °C for 20 h. Assays were carried out in 24-well plates in co-incubation conditions. SEDs were inoculated with a *S. aureus* ATCC 6538 suspension (1 × 107 CFU/mL) supplemented with MELs (final concentrations of 0.5, 1, 2 mg/mL) or SDS (final concentration of 0.5 mg/mL − negative control of growth) or PBS (as positive control of growth) and incubated at 37 °C for 24–48 h. SEDs were, then, washed twice. The inhibitory activity of MELs against *S. aureus* biofilm formation was quantified by means of crystal violet, CV [23]. Briefly, for the determination of the total biomass, biofilms were stained with CV solution (0.2%) for 10 min. CV amount was dissolved with acetic acid (33% in water). Absorbance at 570 nm was measured in each well (Victor3VTM, Perkin Elmer, Italy). In addition, a qualitative analysis of biofilms was also performed by scanning electron microscopy, SEM [24]. Biofilm were fixed with a 2.5% glutaraldehyde in 0.1 M HEPES buffer at 4 °C for 24 h, washed twice in distilled water and dehydrated with EtOH solutions (70%, 90% and 100%). SEDs were then glued to SEM sample holder by double bonding carbon tape and gold sputtered. SEM analyses were conducted in a FEI QUANTA 200 (Fei-Eindhoven, Netherlands) with a variable range 1–30 kV beam voltage. Experiments were performed in triplicate and repeated three times. One-way ANOVA followed by Tukey post hoc test was carried out by means of the statistical R program.

### Metabolic Activity Assay

For the determination of cell metabolic activity, biofilms were dipped in 0.3% 3-(4,5-dimethylthiazol-2-yl)-2,5-diphenyltetrazolium bromide (MTT) solution supplemented with 0.1% glucose and 10 μM menadione. After 30 min of incubation at 37 °C in static conditions, formazan crystals into biofilms were dissolved with DMSO/0.1 M glycine buffer (pH 10.2) solution (7:1). Absorbance at 570 nm (*A*_570_) of the solutions was measured.

### Citrate Synthase (CS) Activity Assay

The assay used in this study was originally described by Shepherd and Garland [25]. This assay measures the production of coenzyme A through a reaction with 5, 5′-dithio-bis (2-nitrobenzoic acid) (DTNB). For the biofilm formation, TSB 50% medium (100 μL/well) was inoculated (1:50) with an overnight TSB-grown culture (30 °C) of *S. aureus* into a 96 well plates. A 20 μL of each sample (cells treated with PBS 1×, MELs 1 mg/mL, 2 mg/mL and adenosine triphosphate 2 mg/mL as an inhibitor of CS) was added to two analogous cuvettes containing at final concentration 0.1 mM acetyl-coenzyme A, 0.2 mM DTNB in 100 mM Tris buffer (pH 8.0) + 1 g/L Triton X-100. The final volume in each cuvette was 1 mL. Samples were then gently mixed and inserted into the Uvikon 941 spectrophotometer (Northstar Scientific, Potton, UK). Duplicate cuvettes were placed into either the reference or sample compartment. The reaction was started by the addition of 10 μL 20 mM oxaloacetate to the sample cuvette. The reaction was measured at 412 nm for 5 min at 30 s intervals for 5 min at + 30 °C. Absorbance was converted to molar concentration and calculated using Beer–Lambert law. The extinction coefficient of DTNB is 13.6 × 10^3^ M^−1^/cm (path length 1 cm, total volume 1 mL). Linearity of CS activity (*R*^2^ = 0.999) was quantified to further validate the method. Total protein content of each sample was measured using a commercially available modified Lowry Assay (Bio-Rad, Hertfordshire, UK). CS activity was normalized to total protein to check its validity as a normalizing factor and to give an estimate of mitochondrial enrichment [26].

### OxoPlate® Assay for Biofilm Formation

For the biofilm formation assay, TSB 50% medium (100 μL/well) was inoculated (1:50) with an overnight TSB-grown culture (30 °C) into the OxoPlate OP96C® as described previously [27]. After 24 h, each biofilm was rinsed twice with PBS 1×, and different concentrations (0.5, 1 and 2 mg/mL) of MELs were added to each well during 30 min at 30 °C. The oxygen concentration in each well was followed for 3 h at 10-min intervals. Fluorescence of each well was measured in dual kinetic mode. Filter pair 1 (544/645 nm) detects fluorescence of the indicator dye. The second filter pair (544/590 nm) measures fluorescence of the reference dye. Oxygen concentration as percentage air saturation was calculated for each well by using the following equation:

$$p_{{{\text{O}}_{2} }} = 100 \cdot \frac{{\left( {\frac{{k_{0} }}{{I_{{\text{R}}} }} - 1} \right)}}{{\left( {\frac{{k_{0} }}{{k_{100} }} - 1} \right)}}.$$

### Cytotoxicity Assay

The potential cytotoxicity of MELs was evaluated as follows [28]. Human Keratinocyte Cells (HaCaT) were plated at a cell density of 1 × 104 cells/well in 24-well plates, in Dulbecco’s modified Eagle’s medium high glucose + 4% foetal bovine serum and incubated for 24 h at 37 °C in 5% CO_2_ atmosphere. Medium was then replaced with one containing MELs (final concentrations of 0.5, 1, 2 mg/mL) or SDS (final concentration of 0.5 mg/mL) and cells were incubated for further 24 h. Fifty microliters of the MTT solution (5 mg/mL) were added in each well. Plates were then incubated for 4 h at 37 °C. Formazan crystals were extracted with 200 μL of HCl solution in isopropanol (250 mL of 0.05 M hydrochloric acid in 5 mL of isopropanol) and *A*_570_ was measured. Experiments were performed in triplicate and repeated three times. One-way ANOVA followed by Tukey post hoc test was performed to evaluate the significance of data in comparison to positive and negative controls by using the statistical R program.

## Results

### Rapeseed Oil Induces the Production of MELs

**Fig. 2:**
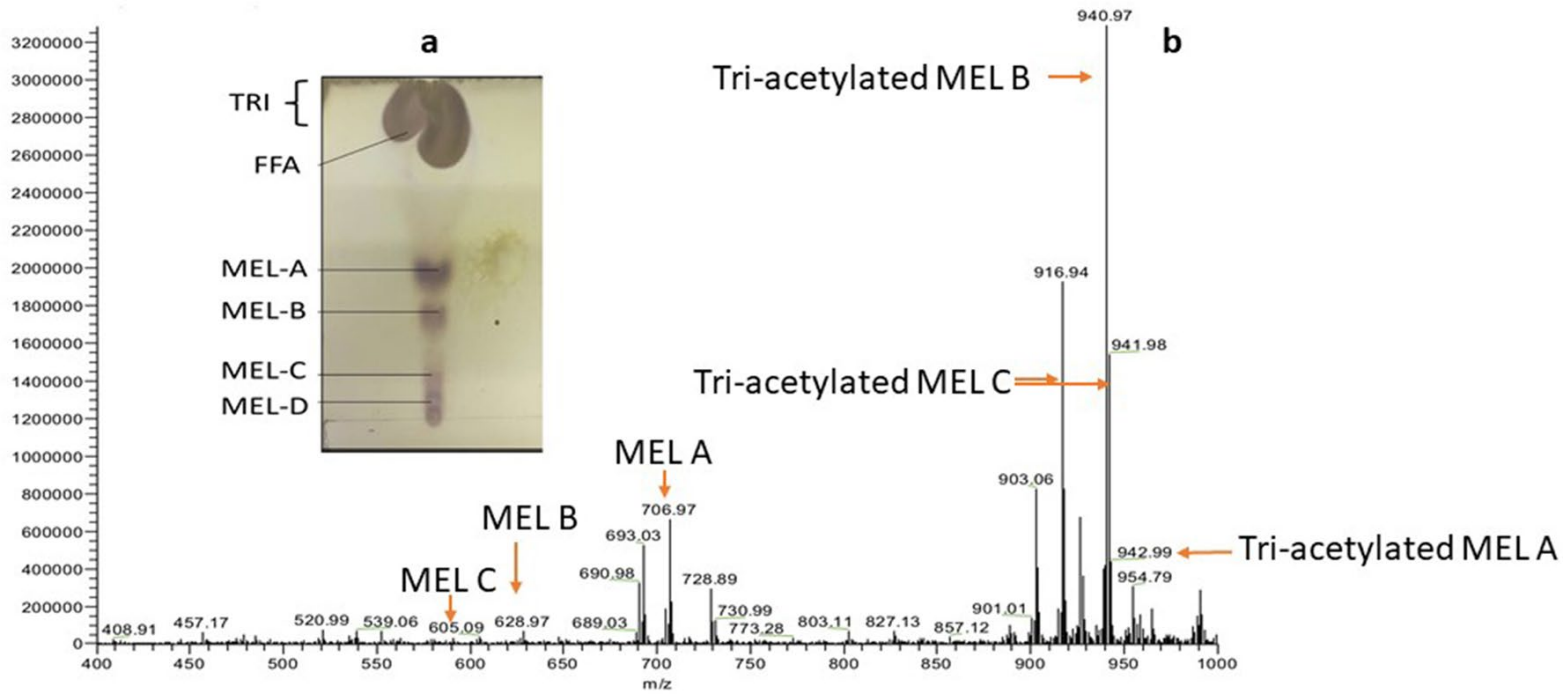
Identification of MELs by different analytical methods. **a** TLC obtained from MELs partially purified (*FFA* free fatty acids, *TRI* tri-acylated MELs). **b** ESI–MS spectrum from MELs partially purified

Production of MELs was carried out through a 5 days fermentation process. During the first stage of the fermentation process the oxygen levels decreased steadily as a consequence of an active growth of *P. aphidis*. The endpoint of the batch phase was after 48 h of fermentation (data not shown). A manual transition to a fed batch was applied, rapeseed oil was added at an initial rate of 4 g/h. Reduced levels of dissolved oxygen limited the production. MELs extracts were analysed by TLC on silica gel 60 TLC plates in two‑step visualization allowing for distinction between oils and fatty acids (Fig. 2a), showing the presence of MELs partially purified, FFAs and tri-acylated MELs (TRI). For the identification of individual isomers (Fig. 2b), a Waters Micromass LCT TOF, with electrospray ionization operated in negative ion mode was used and MEL- A, -B and -C were detected as well as tri-acylated MELs A, B and C according to their *m*/*z*, individual monomers can be seen in Table A (Supplemental Information), where it can be seen that the relative abundance of tri-acylated MELs (A, B, C) is higher than the individual monomers.

### MELs Inhibit Biomass Formation by *S. aureus*

**Fig. 3:**
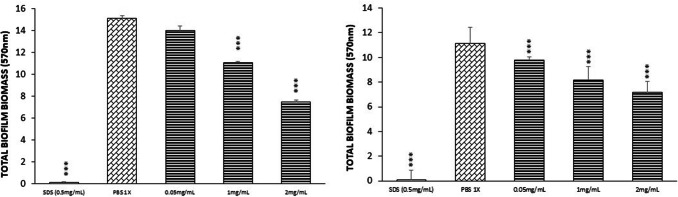
Effect of MELs at different concentration on *S. aureus* ATCC 6538 biofilm formation. **a** 24 h and **b** 48 h, using crystal violet as an indicator. Assays were carried out in triplicate and the experiments were repeated three times (*n* = 9)

The effect of different concentrations of MELs and SDS (as negative control of growth) on *S. aureus* ATCC 6538 biofilm formation was evaluated in terms of total biomass. In general, MELs showed an interesting ability on the inhibition of the development of *S. aureus* biofilms up to 48 h (Fig. 3). In particular, starting from a concentration of 0.5 mg/mL (at 48 h), MELs induced a concentration-dependent reduction of biomass built-up, compared to the control (*P* < 0.001), which can be also seen through SEM in Fig. A (Supplemental Information).

### MELs Reduced Metabolic Activity on *S. aureus* Biofilms

**Fig. 4:**
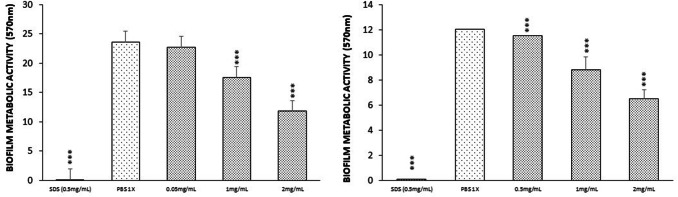
Metabolic activity of *S. aureus* ATCC 6538 biofilms in the presence of MELs at different concentration on at **a** 24 h and **b** 48 h, using MTT as an indicator of metabolic activity. Assays were carried out in triplicate and the experiments were repeated three times (*n* = 9)

Metabolic activity (MTT) and CS assays were performed in order to determine the cell viability of biofilms of *S. aureus*. Over the total 48 h, all MEL co-cultures displayed reduced cell metabolic activity judged by the MTT assay, with all but 0.5 mg/mL displaying a significant difference against the relative controls at 24 h but with a significate effect at the same concentrations at 48 h. Biofilms exhibited continuous reductions in cell viability following the increase in MELs concentration (Fig. 4). A similar trend is observed when the activity of CS was determined on 24 h biofilms treated with 1 and 2 mg/mL of MELs and ATP as a known inhibitor of the tri carboxylic acid cycle CS [29], showing an inhibition on the enzymatic activity of 15, 25 and 40% of inhibition, respectively (Table 1).

**Table 1:** Citrate synthase activity following exposure of MELs at different concentrations

Microorganism and treatment	Activity (nmol/min/mg protein)
*S. aureus* ATCC 6538: Control	2.242
MELs 1 mg/mL	1.967
MELs 2 mg/mL	1.733
ATP 2 mg/mL	1.3452

Biofilms (24 h) were exposed to the treatment for 30 min

### MELs Cause Reduction of Oxygen Consumption in Biofilms of *S. aureus* ATCC 6538

**Fig. 5:**
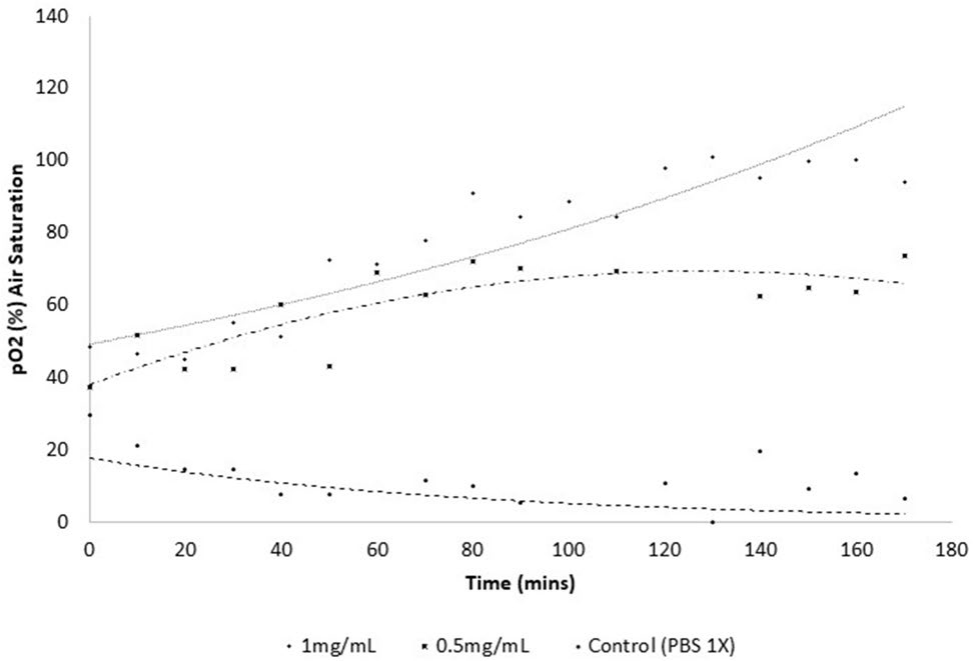
Oxygen consumption of *S. aureus* ATCC 6538 biofilms after 30-min treatment, using different concentrations of MELs. The relative concentration of dissolved oxygen is expressed as the percentage of saturation concentration versus time after addition of the different treatments. Concentrations used are indicated

Biofilms of *S. aureus* ATCC 6538 treated with PBS 1× displayed expected $p_{{{\text{O}}_{2} }}$ levels throughout, showing a stable metabolic activity, due to the continuous lower amount of $p_{{{\text{O}}_{2} }}$ displayed within the system during the 3 h. On the other hand, increased concentrations of MELs lead to an increase in air saturation, reflected again by increased levels of $p_{{{\text{O}}_{2} }}$. The 1.0 mg/mL of MEL displayed the most significant effect on $p_{{{\text{O}}_{2} }}$ and, compared with 0.5 mg/mL, finished with a greater difference in $p_{{{\text{O}}_{2} }}$ than the initially shown. Within the initial 90 min, there are relatively significant fluctuations in $p_{{{\text{O}}_{2} }}$ for both MEL concentrations (Fig. 5), where 0.5 mg/mL show increased $p_{{{\text{O}}_{2} }}$ reductions than 1.0 mg/mL. These fluctuations are clearer in the MELs treatments and are more consistent during the initial 90 min. Despite both MEL treatments exhibiting increased $p_{{{\text{O}}_{2} }}$, the relative progression by which this is achieved seem very different across the two concentrations. This leads to strikingly dissimilar patterns in trend lines for both MEL treatments; 0.5 mg/mL slowly reaches a decline in $p_{{{\text{O}}_{2} }}$, while 1.0 mg/mL displays a relatively sharp rise in $p_{{{\text{O}}_{2} }}$ until the end of the experiment.

### MELs Do Not Induce Side Effects on Mammalian Cells

**Fig. 6:**
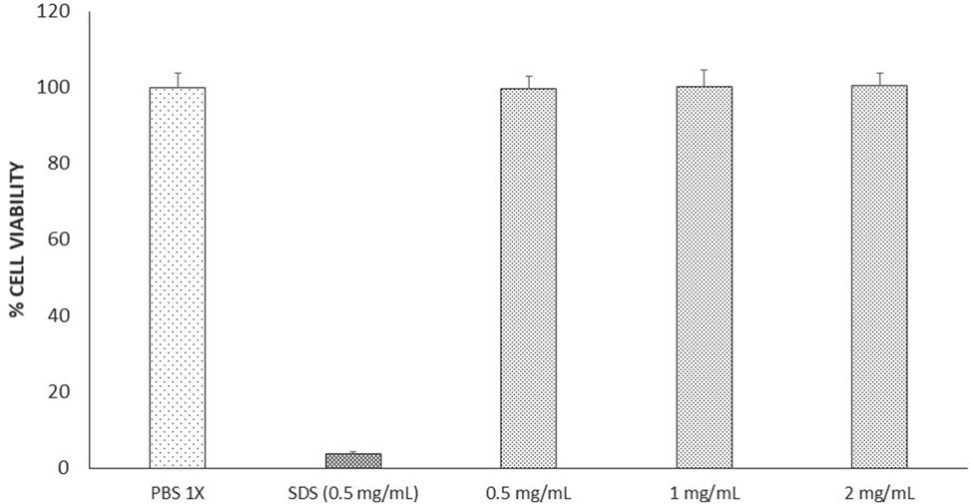
Effect of various concentrations of MELs on HaCaT cell viability. Cell line seeded with different treatments, and viability determined by MTT assay. Positive control higher percentage of viable cells: PBS 1×. Negative control lower percentage of viable cells: SDS 0.5 mg/mL

Results concerning the cytotoxicity evaluation of MELs and SDS are shown in Fig. 6. No side effects were observed when HaCaT cells were expose to MELs concentrations (*P* > 0.05 in comparison with positive control). On the other hand, the exposure to SDS caused the death of almost all of the cell (*P* > 0.05 in comparison with negative control).

## Discussion

Antibiotic resistance is a public health issue worldwide, resulting in a demand for alternative solutions that decrease the resistance of a wide number of gram positive and gram negative microorganisms [30]. Naturally biosurfactants fall under this criteria, simultaneously addressing the potential significance of anti-adhesive and AMR within established or developed biofilms, while exhibiting significant biocompatibility [8, 9]. Understanding the architectural properties that facilitate the formation of biofilms, coupled with the role that biosurfactants have on this process and the means by which AMR is achieved is a fundamental step in determining potential combinations of treatments [5]. The present study determines the effectiveness of various MELs concentrations to promote *S. aureus* ATCC 6538 biofilm disruption, from different physiological standpoints.

MELs displayed significant disruption in biofilm biomass against developed *S. aureus* ATCC 6538 biofilms, at all MEL concentrations tested, showing increased disruptions at higher concentrations (> 1 mg/mL). It is likely that the higher concentrations of MELs result in a degree of biofilm disruption, reflected in the larger OD values obtained by the CV assay, and as consequence the cytoplasmatic content was outpoured after 24 h, pattern that has been previously reported on other Gram + and Gram − microorganisms in the presence of sophorolipids biosurfactants [31, 32]. It is clear that MELs cause similar disruptions to biofilm physiology of *S. aureus* ATCC 6538, without harming Human Keratinocyte Cells as can be seen in Fig. 6.

Cell metabolic activity was quantified using the MTT assay, as it is an efficient method for biofilm metabolic evaluation [14, 22] and the CS enzymatic activity due to its role in the citric acid cycle, where it catalyzes the condensation of oxaloacetate and acetyl-coenzyme A for the formation of citric acid and coenzyme A [14]. Due to the complexity and spatial heterogeneity within biofilm physiological activity, it is difficult to associate reductions in cell metabolic activity to cell death. The persistence of biofilms and formation of diffusion gradients influence this property, as processes such as metabolic dormancy would lead to limited diffusion and respective reductions in metabolic activity [33]. While this would present changes in cell viability, this is a natural step in *S. aureus* biofilm development, within regions of limited nutrients and oxygen, or in the presence of specific antimicrobials. Therefore, reductions in cell metabolic activity do not necessarily result in cell death but reflect that the biofilm is undergoing a process that reduces nutrient intake on the outside of the matrix, optimizing internal processes and the external pumping of antimicrobial agents. There are significant decreases in cell metabolic activity across all concentrations of MELs, with the higher concentrations resulting in larger reductions, a principle shared with disruptions in biofilm biomass.

It is evident that biofilm morphology is heavily influenced by the structural heterogeneity and relative control of gene expression; which causes alterations in the diffusion gradients and the metabolic activity of cells, properties owing to the high virulence and survivability of biofilms [34]. Aerobic biofilms complex structure consists of microbial cell clusters and interstitial voids, which is partly dictated by oxygen distribution and relative transport. These voids facilitate oxygen transport from the bulk liquid into the biofilm, the rate of which is influenced by the biofilm structure itself [35]. A fluorescence assay system (OxoPlates®) was used to quantify oxygen saturation, following overnight growth and subsequent treatment with different concentrations of MELs. The control (PBS 1×) demonstrated a reduction of oxygen within the growth medium, which became more severe at each 10-min interval and showed almost complete depletion by the end. This is to be expected within fully functioning biofilms, as the metabolic and biochemical processes that require these high levels of oxygen are not disturbed. Increased levels of oxygen within the media is indicative of alterations in these processes, while simultaneously reflecting the efficiency of the diffusion gradients and relative distribution of oxygen within the biofilm [33]. This was displayed at both MEL concentrations, suggesting metabolic activity disruptions are taking place, but progressing differently at each concentration. An initial delay is showed at 1 mg/mL, which is not observed at 0.5 mg/mL, followed by a substantial increase that does not reach a steady state. On the opposite side, there is a lack of delay and a relatively early stationary phase of oxygen saturation, displayed by 0.5 mg/mL. This behaviour suggests that the concentrations used act through different mechanisms of action, with 0.5 mg/mL acting as a bacteriostatic compound and 1 mg/mL exhibiting bactericidal properties.

The point at which an antimicrobial agent becomes ineffective, and the relative rate that the disruptions in biofilm composition return to a baseline level, is an essential property of any antimicrobial agent. An understanding of this allows potential treatments that incorporate additional antibiotic compounds, enforcing complete bactericidal effects which would not be present otherwise [27]. Further work should include determining optimal combinations, a factor that will greatly vary between species and the relative stage of development of the biofilm.

## Conclusions

Preventing bacteria from adhering to the surface of medical devices will reduce the frequency of pre- and post-operative infections that could effectively decrease the mortality rate of patients with recurring infections due to antimicrobial resistant pathogens. Interest in the use of biosurfactants in general has steadily increased particularly in the healthcare industry to reduce infections. In this work, we showed that MELs biosurfactants have a positive effect on the disruption of *S. aureus* ATCC 6538 biofilm biomass, reducing the metabolic activity and showing a possible bacteriostatic/bactericidal effect on 24 h mature biofilm. Although the molecular interactions between MELs and *S. aureus* are not yet elucidated, these surface-active compounds show exceptional antimicrobial properties that could be further explore for biotechnological applications.

## Electronic supplementary material

Below is the link to the electronic supplementary material.

Supplementary file1 (DOCX 14 kb)

Supplementary file1 (JPG 89 kb)
