# Supplementary material for: Production of Mannosylerythritol Lipids (MELs) to be Used as Antimicrobial Agents Against S. aureus ATCC 6538
Source: Curr Microbiol. 2020 Mar 2;77(8):1373–80. doi: 10.1007/s00284-020-01927-2 (PMC7334285; doi:10.1007/s00284-020-01927-2)
Supplement: Supplementary file 1 — Supplementary file1 (DOCX 14 kb) [file 284_2020_1927_MOESM1_ESM.docx]

**Table A.** Types of partial fatty acids detected by ESI–MS with corresponding retention times.

| Retention time (sec) | MS [M- H]^+^ | | | | | | |
| --- | --- | --- | --- | --- | --- | --- | --- |
|  | **MEL-A** | **MEL-B** | **MEL-C** | **MEL-D** | **TRI-A** | **TRI-B** | **TRI-C** |
| 12.91 | 684.92 | 661.02 | 627.26 | 606.96 | 962.83 | - | - |
| 19.96 | 690.92 | 640.85 | 626.97 | 605.09 | 938.87 | 889.22 | 885.02 |
| 22.26 | 692.88 | 628.84 | 625.32 | 562.87 | 940.80 | 925.88 | 916.97 |
| 23.89 | 650.23 | 649.16 | 600.75 | 546.90 | 958.84 | 926.90 | 881.05 |
| 25.47 | 682.85 | 647.22 | 632.98 | 580.96 | 968.80 | 944.81 | 930.87 |
| 26.38 | 695.04 | 663.30 | 619.22 | 577.22 | 950.93 | 936.96 | 925.05 |
| 27.29 | 661.13 | 632.86 | 609.94 | 577.23 | 952.96 | 939.14 | 929.04 |
| 28.05 | 663.31 | 635.03 | 621.11 | 558.84 | 981.04 | 927.02 | 909.08 |
| 29.70 | 665.25 | 639.18 | 605.10 | 585.24 | 983.07 | 955.05 | 923.02 |
| 30.95 | 689.22 | 623.21 | 562.90 | 551.15 | - | - | - |
| 32.43 | 681.23 | 594.18 | 567.25 | 551.19 | - | - | - |
| 40.08 | 693.02 | 591.21 | 593.17 | 554.85 | 950.97 | 940.02 | 939.05 |
