# Supplementary figures and images for: Production of Mannosylerythritol Lipids (MELs) to be Used as Antimicrobial Agents Against S. aureus ATCC 6538
Source: Curr Microbiol. 2020 Mar 2;77(8):1373–80. doi: 10.1007/s00284-020-01927-2 (PMC7334285; doi:10.1007/s00284-020-01927-2)

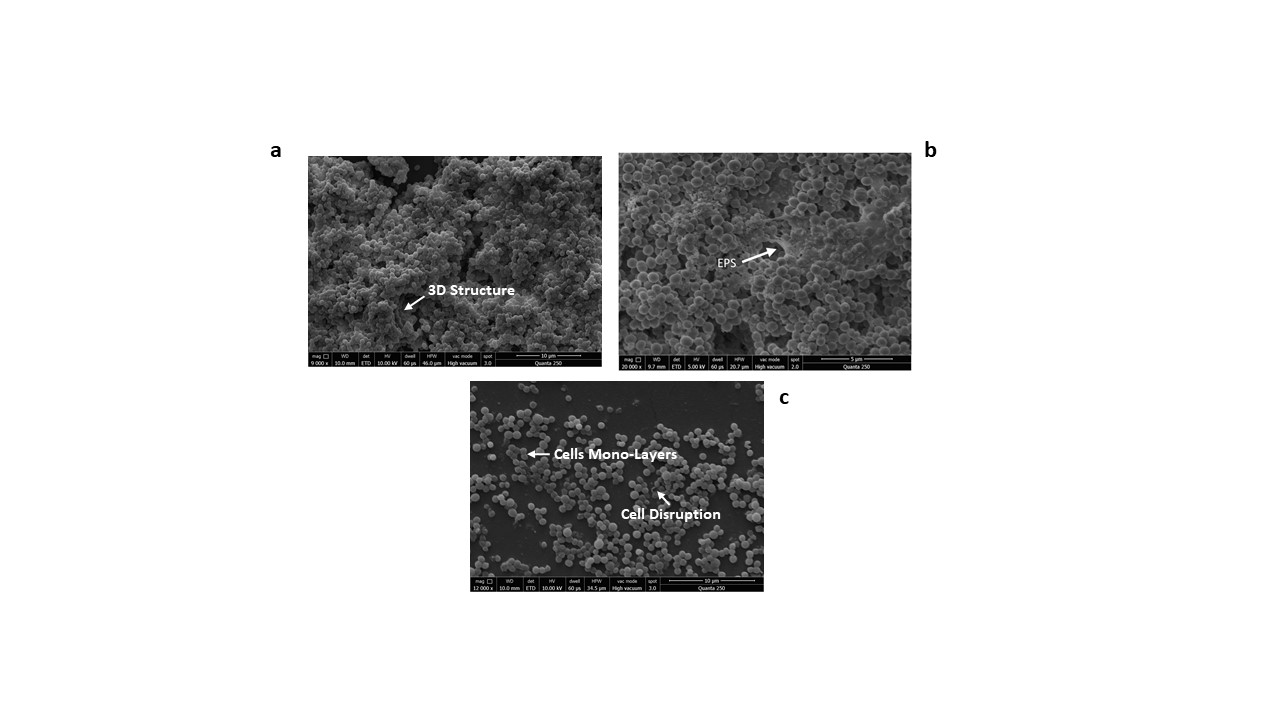

Supplement: Supplementary file 2 — Supplementary file1 (JPG 89 kb) [file 284_2020_1927_MOESM2_ESM.jpg]
